# In Vitro Infant Faecal Fermentation of Low Viscosity Barley β-Glucan and Its Acid Hydrolyzed Derivatives: Evaluation of Their Potential as Novel Prebiotics

**DOI:** 10.3390/molecules24050828

**Published:** 2019-02-26

**Authors:** Ka-Lung Lam, Kin-Chun Ko, Xiaojie Li, Xinxin Ke, Wai-Yin Cheng, Tianfeng Chen, Lijun You, Hoi-Shan Kwan, Peter Chi-Keung Cheung

**Affiliations:** 1 Food and Nutritional Sciences, School of Life Sciences, The Chinese University of Hong Kong, Shatin, Hong Kong; kocaslam@gmail.com (K.-L.L.); ekkcgoo@gmail.com (K.-C.K.); lixiaojie@link.cuhk.edu.hk (X.L.); 1155053989@link.cuhk.edu.hk (X.K.); nanocheng@link.cuhk.edu.hk (W.-Y.C.); hskwan@eservices.cuhk.edu.hk (H.-S.K.); 2 Department of Chemistry, Jinan University, Guangzhou 510632, China; tchentf@jnu.edu.cn; 3 School of Food Science and Engineering, South China University of Technology, Guangzhou 510640, China; feyoulijun@scut.edu.cn

**Keywords:** acid hydrolysis, depolymerization, infant faecal microbiome, short chain fatty acids, ammonia, SCFAs/NH_3_

## Abstract

Barley contains high level of β-1,3-1,4-glucans (BBGs) which can be fermented by microbes and are a potential prebiotic. In the present study, native BBG with low viscosity and a MW of 319 kDa was depolymerized by acid hydrolysis to produce a series of four structurally characterized fragments with MWs ranging from 6–104 kDa. In vitro fermentation of these BBG samples by infant faecal microbiome was evaluated using a validated deep-well plate protocol as parallel miniature bioreactors. Microbial taxa were identified using 16S amplicon sequencing after 40 h of anaerobic fermentation. Bioinformatics analysis including diversity indexes, predicted metagenomic KEGG functions and predicted phenotypes were performed on the sequenced data. Short chain fatty acids and dissolved ammonia were quantified and the SCFAs/NH_3_ ratio was used to evaluate the eubiosis/dysbiosis potential. Correlation analysis showed that most of the parameters investigated showed a parabolic function instead of a monotonous function with the BBG samples having different MWs. Among the five BBGs, it was concluded that BBG with an intermediate MW of 28 kDa is the most promising candidate to be developed as a novel prebiotic.

## 1. Introduction

Oat and barley are widely consumed worldwide in different forms, including direct consumption, ground powders, fermented beverages and so on, with the world oat consumption being 22,777 thousand metric tons and that of barley, 142,581 thousand metric tons in December 2018 [1]. β-1,3-1,4-Glucans (BBGs) are naturally present in oat and barley as hemi-cellulose and they are now being examined as a functional food with prebiotic potential [2]. However, β-glucans differ from well-known prebiotics such as inulin, FOS, GOS due to their high intrinsic physico-chemical property heterogeneity, including differences in molecular weight (MW), glycosidic linkages, degree and length of branching, water solubility, etc. [3]. The MW of β-glucans is one of the important attributes towards their biological function as it affects solubility, viscosity, and enzymatic breakdown efficiency [4]. Currently, high purity (>95%) commercial native β-1,3-1,4-glucans with different MW from barley (BBGs) and oat are available from Megazyme (County Wicklow, Ireland). For example, a total of five different MW β-1,3-1,4-glucans have been used for investigation of their effects on in vitro fermentation using human faecal microbiota [5] and three BBGs of different MW used in in vitro fermentation with porcine faeces [6]. There is also another publication that employed β-1,3-1,4-glucans extracted from barley with different molecular weights but with a lower purity [7]. In this study, we employed low viscosity BBG from Megazyme and its acid hydrolyzed derivatives, with reported purity >95%.

In the present study, we aimed at evaluating the enhancement of the in vitro infant faecal fermentation of BBG with lower MW than the native ones produced by controlled acid hydrolysis. 16S amplicon profiling was determined to assess the microbial composition/diversity. Human infant faecal samples were chosen due to the high viscosity and texture similarity when in a slurry form with in vitro culture medium. Microbial fermentation is catalytically multi-fold, including saccharolytic, proteolytic, lipolytic types and so on [8]. In this study, BBGs of different MW were added as sole carbon sources to the culture medium, commonly containing partially digested proteins. Therefore, saccharolytic and proteolytic fermentation are mainly involved in this investigation. The effect of polymeric β-glucans and its monomer glucose on microbial profile was also compared and the correlations and insights from the phenotypic data, microbial profile and MWs of BGGs were discussed.

## 2. Results

### 2.1. Chemical Composition and Structual Characterization of BBG Samples

All BBG samples were predominantly carbohydrate in nature (97–98% by weight), with glucose as the only monosaccharide and contained only trace amounts of protein (less than 1% by weight) without significant difference (Table 1). The monosaccharide profile of native BBG and depolymerized BBG_0.2(2) shown in Figure S1A indicated that the only sugars found were glucose and allose (internal standard). The FT-IR spectra (Figure S1B) and the proton NMR spectra (Figure S1C) confirmed the chemical structure of BBG and BBG_0.2(2) indicated that acid hydrolysis did not introduce any structural changes to the functional groups of the BBG derivatives. Increasing acid concentration and time of hydrolysis reduced the MW of BBG derivatives as shown in Table 1.

**Figure 1:**
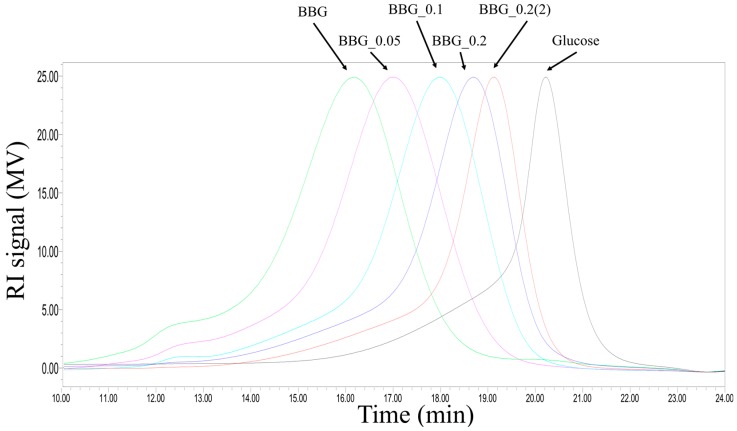
SEC chromatograms showing the MW distribution of BBGs and its depolymerized derivatives. Glucose was used as a monomer control.

All depolymerized BBG derivatives had a MW higher than the glucose monomer with BBG_0.2(2) having the lowest MW of 6 kDa as shown in Figure 1. Linkage analysis of methylated BBG samples by GC-MS indicated the presence of three major partially methylated alditol acetates (PMAAs) including 2,3,4,6-Me_4_-Glc (terminal glucose), 2,4,6-Me_3_-Glc (1,3-linked glucose) and 2,3,6-Me_3_-Glc (1,4-linked glucose) (Table 2). These linkage results were consistent with the typical mixed-1,3-1,4 linkages found in BBG. Based on the molar ratios of these three PMAAs, the change in the ratio of 1,3- and 1,4-glycosidic linkages before and after various acid hydrolysis was approximately 1:6, suggesting that the acid hydrolysis has no preference for β-1,3 or β-1,4 linkages (Table 2). In conclusion, controlled partial acid hydrolysis is an effective means of MW reduction in BBG.

### 2.2. BBG In Vitro Fermentation Using Infant Faecal Inocula

#### 2.2.1. Fermentation of BBG Samples Evaluated by Total Bacterial Count and Total DNA Extracted

Results of the bacterial total plate count (TPC) in terms of colony formation units (CFUs) and the fold of population increase relative to glucose are shown in Table 3 and a representative plate of each of the five BBG samples and glucose monomer is shown in Figure S2. In the present results, the concentration of the extracted microbial DNA was accessed by Nano-drop (Thermo Scientific, Waltham, MA, USA) and the fold changes of the five BBG samples relative to glucose are shown in Table 3. All BBG samples supported the growth of more microbes than glucose, which was probably due to the larger amount of monomeric glucose produced after enzymatic hydrolysis of the BGG samples on molar basis. TPC results indicated that BBG had the highest CFUs (3.7-fold increase), followed by BBG_0.2 (2.9-fold), BBG_0.1 (2.3-fold), BBG_0.2(2) (2.2-fold), and BBG_0.05 (1.0-fold) when compared to glucose. Total DNA results indicated that the increase of biomass was in a descending order of: BBG_0.1 (4.2-fold), BBG_0.05 (3.9-fold), BBG_0.2 (3.6-fold), BBG (3.4-fold) and BBG_0.2(2) (2.8-fold). There seemed to have a nonlinear relationship of increase in microbial populations with a decrease in MW of the BGG samples. The discrepancy between the results of TPC and total DNA might be explained by the fact the TPC is the final outcome of complex interactions of all microbes including self/mutual promotion and/or inhibition which might not only depend on the MW of the carbon source.

Compared to TPC, the amount of total DNA extracted in a microbial fermentation could better reflect the total bacterial biomass supported by the carbon source since it is not selective and medium-independent [9]. Therefore, DNA concentration was selected to represent total bacteria count of samples in the following analysis.

#### 2.2.2. Short Chain Fatty Acids and Dissolved Ammonia Content after In Vitro Fermentation

**Figure 2:**
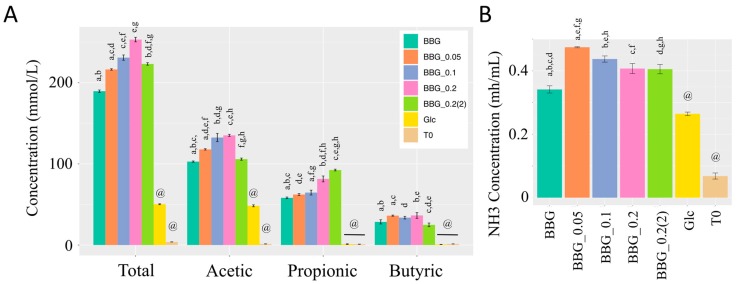
(**A**) Short chain fatty acid profiles and (**B**) ammonia concentration of the five BBG samples and glucose monomer. Different superscripts (^a–h^) represent significant difference by one-way ANOVA (Tukey HSD post-hoc test), *p* < 0.05. @: denotes statistically significant different from all other groups.

The highest level of short chain fatty acids (SCFAs) produced was found in BBG_0.2 which was five times that of glucose as shown in Figure 2A. Glucose only generated acetic acid and its concentration was lower than all the five BBGs. This might be explained by the fact that infant faecal inoculum contained a myriad of bacteria that not only converted glucose into SCFAs, but also other metabolites. Acetic acid was the major SCFAs produced by all BBG samples, followed by propionic acid and then butyric acid. The distribution of SCFAs produced by different BBG samples also varied. The highest acetic acid production was found in BBG_0.1 and BBG_0.2, while BBG_0.2(2) having the lowest MW had the highest concentration of propionic acid (Figure 2A).

The highest ammonia production was obtained by BBG_0.05 samples with original BBG produced the least among the five BBGs (Figure 2B). All BBGs produced more ammonia compared to glucose. As shown in Figure 2A, glucose produced the highest level of propionic acid, which might explain its least ammonia production as propionate ion was shown to be a growth inhibitor to pathogenic/spoilage microbes [10].

### 2.3. Microbiome Profile Changes after BBG Fermentation

#### 2.3.1. Change of Infant Faecal Microbiome Profile by Fermentation of BBG Samples

**Figure 3:**
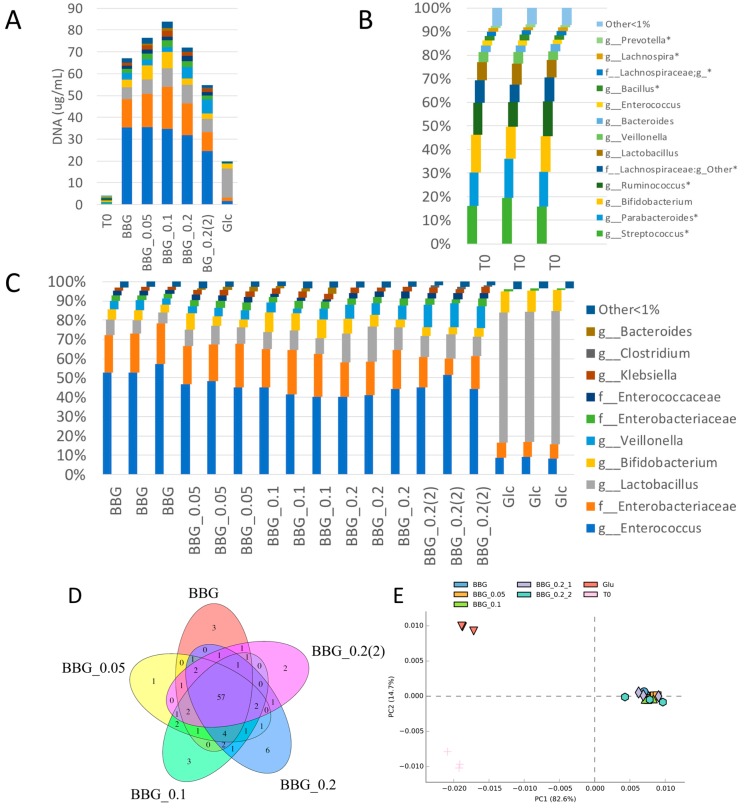
16S amplicon sequencing results of fermentation of BBG samples by infant faecal inoculum. (**A**) Bar plot of the major identified bacterial taxonomy scaled to the total extracted DNA concentration, noted as quasi-absolute bacterial amount; (**B**) Enlarged view of the relative abundance of the taxon >1% of T0 group at the beginning of fermentation; (**C**) Enlarged view of the relative abundance of the taxon >1% of the five BBG groups and glucose monomer, Glc; (**D**) Venn diagram showing the similar and different number of bacteria taxonomy identified; (**E**) β-diversity analysis showing the principle component analysis of the five BBGs and glucose monomer.

The microbial taxonomy profiles of the five BBG samples, glucose and time-0 (T0) are shown in Figure 3A with abundance data scaled according to sample total DNA concentration, reflecting a quasi-absolute taxa amount. 

It was clearly shown that T0 group had the least taxa abundance in the beginning. Moreover, the biological triplicates among each group gave similar microbiome profiles, demonstrating a sample size of three could be sufficient for gaining some insights. Figure 3B showed the relative abundance of T0 group with distinct taxa (average relative taxa abundance >1%) compared to the BBGs samples. Taxa unique to T0 group were noted with "*" as shown in the figure legend, namely, *Prevotella* sp., *Lachnospira* sp., *Bacillus* sp., other genus in *Lachnospiraceae* family, *Ruminococcus* sp., *Parabacteroides* sp. and *Streptococcus* sp. These taxa abundance contributed less than 1% after BBGs/glucose fermentation. Figure 3C shows that all the BBGs shared similar microbial taxa distribution while glucose monomer though had similar taxa but with a distinctive distribution.

The Venn diagram of the microbiome profiles of the five BBG samples indicates that they all shared 57 core microbial taxa and some group-specific unique taxa (Figure 3D). The number of group-specific taxa ranged from 1 to 6 and these specific taxa (family/genus) were *Actinomycetales*, *Oscillospira*, and *Peptoniphilus* for BBG; *Bacilli* for BBG_0.05; *Cellulomonas*, *Aerococcus* and *Leuconostoc* for BBG_0.1; *Rothia*, *Pediococcus*, *Roseburia*, *Eubacterium*, *Burkholderiaceae*, and *Oceanospirillaceae* for BBG_0.2; *Anoxybacillus* and *Weissella* for BBG_0.2(2). These specific taxa are highlighted in Table S1. All these unique taxa were composed of less than 0.001% of total taxa, implying their minor role in BBG metabolism and SCFAs/NH_3_ production. β-Diversity, depicted in Figure 3E, shows that all the BBG samples were clustered as a group with glucose monomer as a distinctive group and T0 as another distinct group.

**Figure 4:**
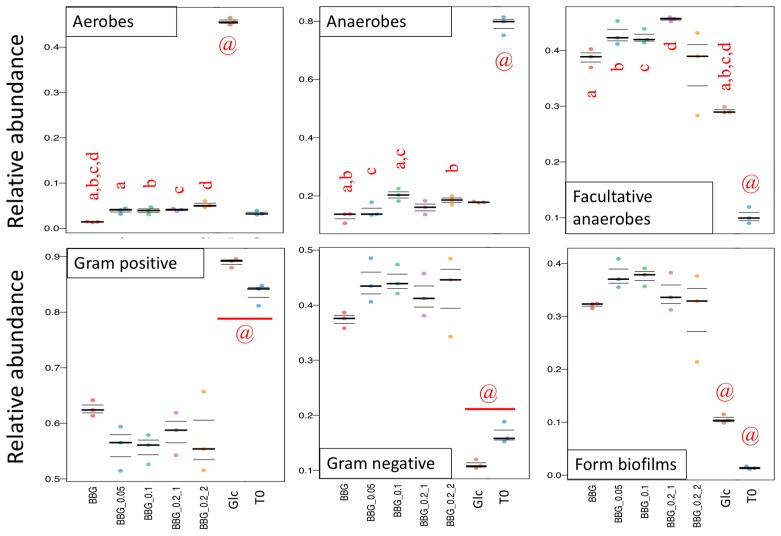
Predicted output of selected phenotype by BugBase. Relative abundance of aerobes, anaerobes and facultative anaerobes are in the upper panel. Gram-positive bacteria, Gram-negative bacteria and taxa that are predicted to form biofilm are given in the lower panel. Significant difference analyzed by one-way ANOVA (Tukey HSD post-hoc test), *p* < 0.05. @: denotes statistically significant different from all other groups.

**Figure 5:**
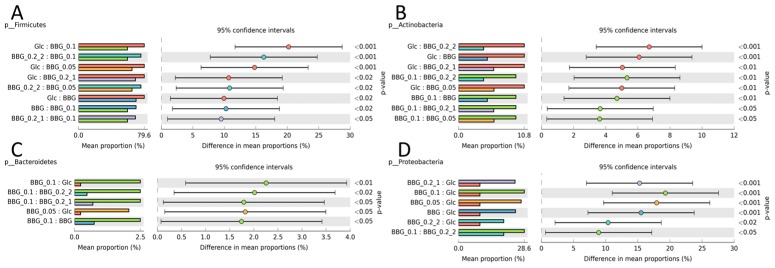
Significantly different taxa in phyla level identified using ANOVA (Tukey HSD post-hoc test) with Benjamini-Hochberg FDR multiple test correction. (**A**) Firmicutes; (**B**) Actinobacteria; (**C**) Bacteroidetes; and (**D**) Proteobacteria. These 4 major taxa accounted for 99.90 ± 0.07% of total taxa identified.

Figure 4 shows the results from BugBase with the upper panel being the predicted relative abundance of aerobes, anaerobes and facultative anaerobes. The relative abundance of aerobes of 5 BBGs were similar to T0 but not the glucose group. As for the predicted anaerobe proportion, T0 group contained the highest abundance. Since BBGs had similar proportion of aerobes with T0 but different proportion of anaerobes with T0, the difference should be contributed by the proportion of facultative anaerobes. As shown, the five BBGs had various proportions of facultative anaerobes with BBG_0.2 having the highest and BBG the least. T0 group had the least proportion of facultative anaerobes among all. The lower panel of Figure 4 refers to the predicted Gram-positive, Gram-negative and proportion of taxa capable of forming biofilms. For the five BBGs, a reduction of proportion of Gram-positive taxa and an increase of proportion of Gram-negative taxa compared with glucose was observed. The predicted phenotype of T0 group was of less interest as shown in Figure 3A, while the quasi-absolute number of all bacteria was very low, thus making the comparison of proportion less biologically meaningful. But interesting phenomena could still be observed in the five BBGs and glucose groups. Figure 5 shows the significantly different phylum of the five BBGs and glucose with descending abundance: Firmicutes (Figure 5A), Proteobacteria (Figure 5D), Actinobacteria (Figure 5B) and Bacteroidetes (Figure 5C). Glucose could significantly provide a higher growth support to Firmicutes and Actinobacteria while BBGs allowed enrichment of Bacteroidetes (well-known for complex polysaccharide breakdown such β-glucans [11]) and Proteobacteria.

**Figure 6:**
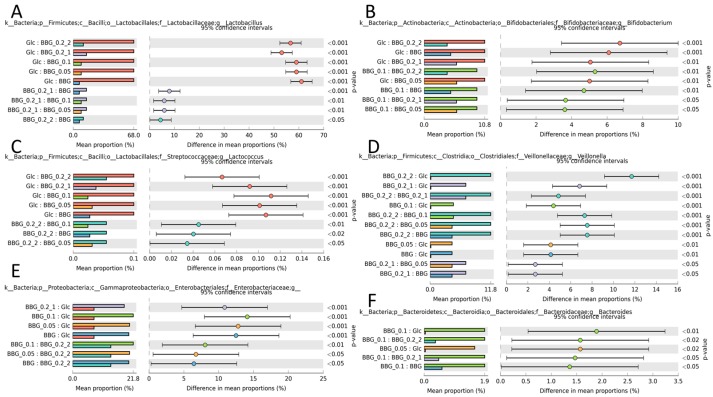
Significantly different taxa in genus level identified using ANOVA (Tukey HSD post-hoc test) with Benjamini-Hochberg FDR multiple test correction. (**A**) *Lactobacillus*; (**B**) *Bifidobacterium*; (**C**) *Lactococcus*; (**D**) *Veillonella*; (**E**) *Enterobacteriaceae*; and (**F**) *Bacteroides*. These taxa were considered beneficial to the hosts.

Figure 6 depicts some host beneficial taxa that were identified in the five BBGs and glucose. Glucose supported more probiotic *Lactobacillus* sp. while low MW BBG out-performed high MW BBG (Figure 6A), a similar phenomenon was also observed in *Lactococcus* sp. (Figure 6C). Another probiotic *Bifidobacterium* sp. was more selectively enriched by glucose than BBGs but among the five5 BBGs, intermediately MW such as BBG_0.1 was more favorable (Figure 6B). On the other hand, BBGs showed better support towards *Veillonella* sp. (higher efficiency in propionate production [12], Figure 6D), *Enterobacteriaceae* family (common enteric microbe in animal gut, Figure 6E), and *Bacteroides* (boom with diet rich in complex polysaccharides, Figure 6F).

**Figure 7:**
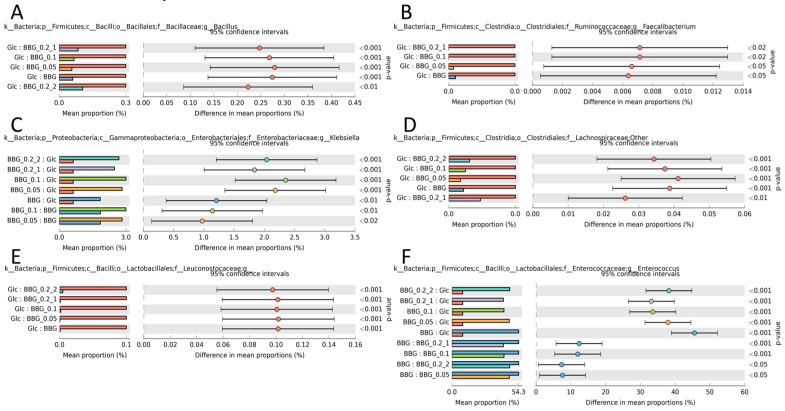
Significantly different taxa in genus level identified using ANOVA (Tukey HSD post-hoc test) with Benjamini-Hochberg FDR multiple test correction. (**A**) *Bacillus*; (**B**) *Faecalibacterium*; (**C**) *Klensiella*; (**D**) *Lachnospiraceae*; (**E**) *Leuconostocaceae*; and (**F**) *Enterococcus*. These taxa were considered either neutral or opportunistic pathogenic to the hosts.

On the contrary, Figure 7 depicts some neutral/opportunistically pathogenic taxa. Among the selected, BBGs reduced *Bacillus* (Figure 7A), *Faecalibacterium* (Figure 7B), *Lachnospiraceae* (Figure 7D) and *Leuconostocaceae* (Figure 7E). However, BBGs augmented the growth of *Klebsiella* (Figure 7C) with some of its species regarded as emerging pathogens [13]. Figure 7F shows *Enterococcus* as the most abundant taxa, commonly found in animal intestines.

Table 4 shows one phylogeny-based α-diversity index (PD whole tree) and 5 non-phylogeny-based indices including Fisher alpha, Berger-Parker d, Shannon, Simpson evenness, and Simpson reciprocal. Briefly, BBG_0.1 showed significantly higher α-diversity than BBG for PD whole tree, Shannon, Simpson evenness, and Simpson reciprocal. While BBG_0.2(2) showed significantly higher value than BBG in terms of Fisher alpha, BBG had a significant higher value of Berger-Parker-d index. Overall, BBG_0.1 was shown to have the highest increase in α-diversity.

#### 2.3.2. Metagenome Prediction of KEGG Functional Annotation

Kyoto Encylopedia of Genes and Genomes (KEGG) functional annotation was done using PICRUSt with standard analysis including 16S copy number normalization and whole metagenome prediction with raw data output are shown in Table S2. The NSTI scores of PICRUSt metagenome prediction for the BBG samples were: BBG (0.032); BBG_0.05 (0.032); BBG_0.1 (0.032); BBG_0.2 (0.033); BBG_0.2(2) (0.031); and that of Glucose (0.034) T0 (0.0589). An NSTI score of 0.03 indicates that the average microbe in your sample can be predicted using a relative from the same (97%) species [14]. All BBG samples had higher number of predicted genes than that of glucose with BBG_0.05 having the highest number (Table S2). “Metabolism" was the highest class of annotated genes in Level 1 as shown in Figure S4A, followed by "Environmental information processing" and “Genetic information processing”. Most genes annotated in “Metabolism” at Level 2 were related to “Carbohydrate metabolism”, followed by “Amino acid metabolism” and “Energy metabolism” with “Lipid metabolism” ranked the sixth as shown in Figure S4B. All BBGs had a higher proportion of predicted KEGG class of "Carbohydrate Metabolism" than glucose monomer and T0, reflecting extra machinery was required to breakdown and absorb β-glucans. Since BBG metabolism, short chain fatty acid production and ammonia production were our targets of investigation, related KEGG classes were single out for more in-depth study using the Statistical Analysis of Metagenomic Profiles (STAMP) approach.

Figure S5A shows the “Carbohydrate metabolism” KEGG class. All BBG samples had a significant increase in the number of predicted genes in carbohydrate metabolism, compared to glucose. The five BBG groups also have higher proportion of predicted KEGG class ABC Transporter (Figure S5B), Bacterial Secretion System (Figure S5C) and Glycan biosynthesis and metabolism (Figure S5D). All the four predicted KEGG classes actually coordinated among themselves, such as secretion system to secrete extracellular enzymes, ABC transporters to uptake carbon/glycan with the expense of cellular energy and glycan metabolism to breakdown complex glycan or use the carbon source for bacterial glycan formation. 

Although “Fatty acid metabolism” does not only involve SCFAs, this class of keys could also be used to infer the potential in SCFAs production. Among the five BBG samples, BBG_0.1 had the highest number of predicted genes annotated to “Fatty acid metabolism”, followed by BBG_0.05, BBG_0.2, BBG_0.2(2) and the native BBG having the lowest number (Figure S6A). Glucose had a similar proportion of predicted genes in fatty acid metabolism with BBG_0.2.

Not only with saccharolytic potential and lipolytic potential, we also extracted some ammonia production related KEGG classes. For example, BBGs stimulated less “Protein digestion and absorption” with medium MW BBG such as BBG_0.005 and BBG_0.1 being more favorable (Figure S6B). However, the BBG groups had a significant higher relative abundance of KEGG classes “Amino acid metabolism” (Figure S6C) and "Nitrogen metabolism" (Figure S6D).

#### 2.3.3. Microbial Biomarkers Identification

**Figure 8:**
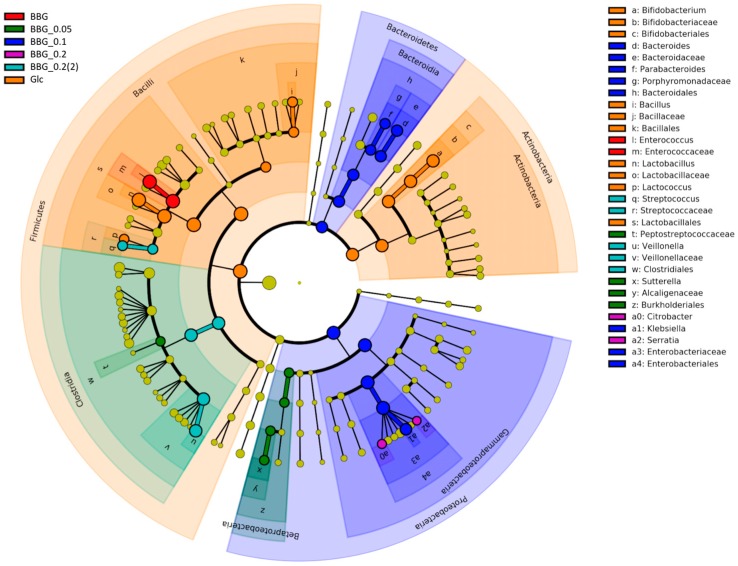
Cladogram of biomarkers identified in the infant faecal fermentation of 5 BBG samples against glucose monomer using LEfSe.

Microbial biomarkers were identified using the LEfSe. Table S3 shows the raw data output of significant biomarkers identified with the five BBG samples and glucose. Briefly, glucose had the highest number of biomarkers compared to the five BBG samples, including *Lactobacillus* and *Bifidobacterium*, while BBG_0.1 gave the highest number of biomarkers among the 5 BBGs, including *Proteobacteria* and *Bacteroides*. Figure 8 shows the cladogram of biomarkers with *Bacteroides*, *Parabacteroides*, *Bacillus*, *Enterococcus*, *Lactobacillus*, *Veillonella*, *Sutterella*, and *Klebsiella* being the dominant ones. LEfSe analysis presented some statistically significant biomarkers, from which we choose some biologically significant taxa for further comparative analysis. Among these biomarkers, excluding the group-specific unique taxa in the Venn diagram analysis (Figure 3D), some interested taxa were chosen for correlation analysis with BBG samples having different MWs.

## 3. Discussion

### 3.1. Efficacy of Controlled Acid Hydrolysis in MW Reduction

One of the major focus of this investigation was to investigate the effects of MW reduction in BBG on microbial fermentation. We have showed that acid hydrolysis in a controlled manner could produce BBG with reduced molecular weight (Figure 1, Table 1 and Table 2) without the modification of functional groups or glycosidic linkages. Controlled acid hydrolysis was also employed for the MW reduction of other β-glucans such as β-13,16-glucans [15]. Acid hydrolysis of β-1,3-1,4-glucan into smaller fragments had also been conducted with different acids, with results indicated that H_2_SO_4_ produced the least glucose monomer and the highest proportion of oligosaccharides than TFA while HCl produce only monomers at low concentration (0.1 or 0.05 M) [16]. Thus, H_2_SO_4_ generates the production of a wider spectrum of smaller fragments. As shown in Figure 1, a lateral transverse of peaks from high MW to low MW BBGS and then to glucose monomer was observed in the size exclusion chromatography (SEC). Although the peaks of the five BBGs only differed in MW, they all had similar peak height, skewness and kurtosis. Therefore, we speculate that acid hydrolysis is a ”gain-by-loss” method, i.e., lower MW samples were obtained by random hydrolysis with steric advantage from both ends. Furthermore, tiny fragments such as mono-, di-, tri-, tetramers, might be gradually released and removed by dialysis. Therefore, controlled acid hydrolysis might not be very efficient in producing very short β-glucans fragments such as oligo-β-glucans which could pass through dialysis tubing or membrane easily. On the contrary, enzymatic degradation using glycosidic linkage/chain-length specific enzymes could selectively cut the polymeric chains into fragments to produce oligomers instead of monomers [17,18]. Therefore, controlled acid hydrolysis serves an excellent purpose of reducing the MW of β-glucans and other polysaccharides extracted from raw materials down to the MW cut-off of tubing/membranes before further reduction into oligomers.

**Figure 9:**
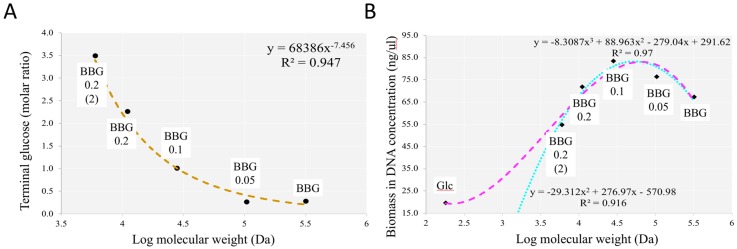
Correlation analysis of selected parameters against BBGs of different MWs with glucose as monomer. (**A**) Terminal glucose molar ratio identified via linkage analysis using GC-MS; (**B**) total biomass support by BBGs and glucose. DNA concentration was used to reflect the quasi-absolute bacterial biomass.

We have tried to correlate different parameters with the MWs of depolymerized BBG samples and glucose (180 Da) as the monomer of BBGs. In Figure 9A, we observe an increase of terminal glucose with MW reduction in log scale. The correlation could be well fitted by a power equation with an R^2^ value = 0.947. Since the 1-3 and 1-4 linkage has an approximate ratio of 1:6, therefore, with this power equation, we could extrapolate the linkage of BBGs with further MW reduction. On the other hand, Figure 9B shows total biomass (bacteria) after 40-hour fermentation with different MW of BBGs. We could observe an increase of biomass as MW was increased due to more compact and dense energy provided by the longer chain β-glucan and then a decrease as higher requirement of energy/time/machinery in breakdown the long compact chain into smaller unit for absorption. Thus, we observe a turning point with MW close to BBG_0.1 (28 kDa). BBG_0.1 allow the most carrying capacity in equal mass of carbon source added.

### 3.2. Polymeric BBGs and Monomeric Glucose on Microbial Taxa

**Figure 10:**
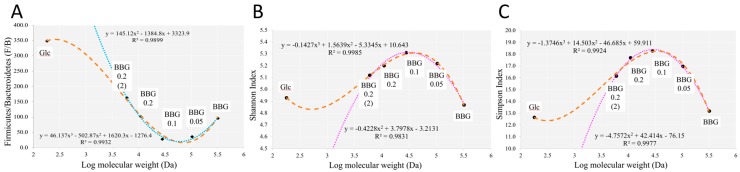
Correlation analysis of selected parameters against BBGs of different MWs with glucose as monomer. (**A**) Firmicutes/Bacteroidetes ratio; (**B**) Shannon α-diversity; (**C**) Simpson α-diversity.

Glucose (Glc) was added as a sole carbon source for the evaluation of monomeric and polymeric glucose chain. Some selected taxa among the five BBGs and glucose have been depicted in Figure 5, Figure 6 and Figure 7. Here, we evaluate the correlation of Firmicutes/Bacteroides (F/B) ratio as one of the indicators in microbiome of gut eubiosis and dysbiosis. It was proposed that a lower of F/B ratio boost eubiosis and promote resistance to certain syndromes. In Figure 10A, we observed a U-shape pattern of F/B ratio against the increase of MW. BBG_0.1 had the lowest F/B ratio among the five BBGs and glucose monomer. Actually, glucose had the highest F/B ratio, in accordance to previous reports, showing high glucose/fructose consumption would lead to increase of F/B ratio in mice without body weight changes [19]. Figure 10B,C show two common α-diversity indexes including the Shannon index and Simpson index. Shannon index emphasizes on both abundance and evenness, while the Simpson index is a dominance index because it gives more weight to common or dominant species [20]. A similar pattern between the two index and the most diversifying carbon source was found in BBG_0.1 that gave peak value in both indexes with an inverted-U shape being observed. 

### 3.3. Effect of Polymeric BBGs and Monomeric Glucose on Saccharolytic and Proteolytic Profiles

**Figure 11:**
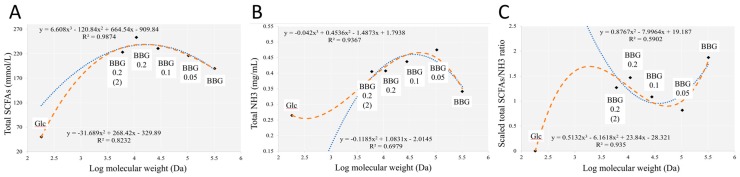
Correlation analysis of selected parameters against BBGs of different MWs with glucose as monomer. (**A**) Total short chain fatty acids (SCFAs); (**B**) total dissolved ammonia (NH^3^); (**C**) SCFAs/NH_3_ ratio after data scaling to both using the min-max approach.

Correlation analysis of the MW of BBG samples together with glucose shows a parabolic function with total short chain fatty acids with the highest concentration found at a MW closed to BBG_0.2 (11 kDa) (Figure 11A), showing BBG_0.2 had the highest saccharolytic potential among the five BBGs though this relationship was not linear. The culture medium (mMCB) used in fermentation contained partially digested protein such as peptone, tryptone, yeast extract and soy peptone. There have been publication showing that more saccharolytic microbial activity and less proteolytic activity confer eubiosis [21]. Moreover, this inverted-U shape pattern was also found in a similar article using oat β-1,3-1,4-glucans of six different MW with the original β-glucan and the lowest MW fraction gave the least total SCFAs production [22]. We have also investigated the concentration of dissolved ammonia after 40-h fermentation (Figure 11B). Generally, hydrolyzed BBGs produced more dissolved ammonia with the native BBG and glucose monomer having similar concentration. Here, we denoted the SCFAs/NH_3_ ratio as an indicator to evaluate the saccharolytic and proteolytic potential. Since the total SCFAs and NH_3_ data are in different scales, both data were first scaled using the min-max approach before their ratio was taken. As shown in Figure 11C, glucose monomer gave the least SCFAs/NH_3_ ratio while the native BBG gave the highest SCFAs/NH_3_ ratio, followed by BBG_0.2. The use of SCFAs/NH_3_ ratio allowed signal to be observed that could otherwise not be found in Figure 11A,B. Eubiosis could be achieved by promoting more saccharolysis or by diminishing the proteolysis. Therefore, we proposed that SCFAs/NH_3_ ratio could serve as a better indicator than just total SCFAs alone.

### 3.4. Insights from the Phenotypic Data, Microbial Profiles against MW Reduction of BBGs

With sufficient carbon sources, microbes can grow better in substrates with lower MW because of the extra energy/time spent in high MW polymeric breakdown and absorption. Monosaccharides such as glucose, and simple sugars such as lactose and sucrose are therefore widely used as sole carbon sources in microbiological culture media preparation. However, the advantage of MW reduction does not follow a linear trend as with MW as demonstrated by Figure 9, Figure 10 and Figure 11. It is because fermentation in pure bacterial culture does not consider the interactions between microbes. For example, although strain specific, the in vitro fermentation of oat β-1,3-1,4-glucan and its hydrolyzed fragments with pure probiotic culture *Lactobacillus helveticus*, *Lactobacillus rhamnosus* and *Bifidobacterium longum* showed that growth and total SCFAs production was highest with glucose, followed by hydrolyzed derivative and then the original oat β-glucan [23]. Important elements of research in microbial community ecology include the analysis of functional pathways for nutrient resource and energy flows, mechanistic understanding of interactions between microbial populations and their environment, and the emergent properties of the complex community [24]. Microbial community interaction is multi-fold, including competition, mutualism, inhibition, promotion and so on. Therefore, the use bacterial community in fermentation leads to a diverse results. In the present study, four very different correlation patterns resulting from fermentation with BBGs with different MWs were extracted and discussed as examples. 

**Figure 12:**
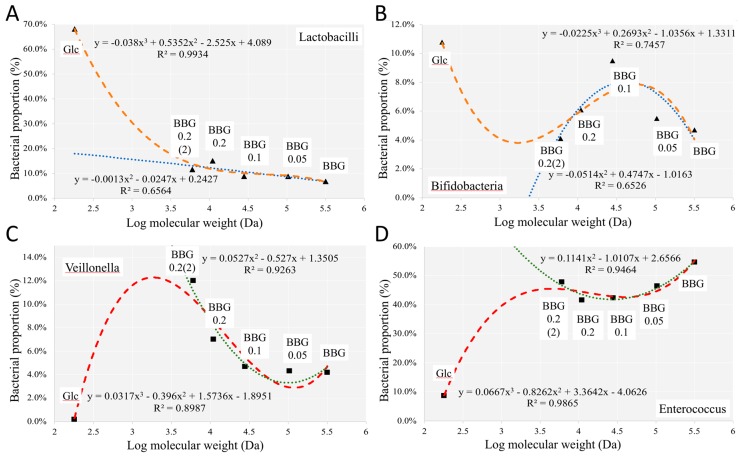
Correlation analysis of selected taxa at genus level against BBGs of different MWs with glucose as monomer. (**A**) *Lactobacillus*; (**B**) *Bifidobacterium*; (**C**) *Veillonella*; and (**D**) *Entrococcus*.

Reduction of the MW in BBGs seemed to increase the population of *Lactobacillus* gradually with glucose having the maximum increase (Figure 12A). Though it was not linear, a monotonous trend was still observed. Such trend suggested that further reduction in the MW of BBGs would probably increase the population growth of *Lactobacillus* among the whole microbial community. On the other hand, Figure 12B shows the pattern of *Bifidobacterium*, with the highest population increase in *Bifidobacterium* being with BBG_01. An inverted-U shape pattern was also recognized among the five BBGs. With reference to glucose, we expected a further reduction in the MW of BBG samples would firstly reduce the population of *Bifidobacterium* followed by an increase again. Actually, glucose monomer induced particularly *Bifidobacterium* growth (a little bit more relative abundance than BBG_0.01). *Veillonella* might have an exceptional ability in fermenting BBG with MW lower than BBG_0.2(2) as shown in Figure 12C. *Veillonella* is a group of microbes well-known for its ability to convert lactate into acetate and propionate during fermentation [12]. This might also contribute to the trend in propionic acid production (Figure 2A) as the growth pattern of *Veillonella* matched well with the propionic acid production pattern. On the contrary, the growth pattern of *Veillonella* was a mirror reflection of *Bifidobacterium* with *Veillonella* having a slight phase difference. This could possibly explain the reduction of *Bifidobacterium* with BBG having a MW lower than BBG_0.2(2) (6 kDa) as *Veillonella* might be able to utilize BBGs with a MW lower than 6 kDa. Figure 12D shows the correlation pattern of the *Enterococcus* which contributed about 60% of total bacterial abundance in all the five BBG samples. It was observed that the population of *Enterococcus* had a moderate increase when the MW was above that of BBG_0.2(2) but decreased drastically when the MW was below that of BBG_0.2(2) (Figure 12D). Growth of *Enterococcus* was supported by polymeric BBGs while glucose monomer, and perhaps dimers and oligomers are extrapolated to be less supportive under in vitro condition.

## 4. Materials and Methods

### 4.1. Materials

All chemicals were obtained from Sigma-Aldrich (St. Louis, Missouri, USA) unless otherwise specified. 

### 4.2. Infant Faecal Sample Collection

Sample collection protocols were approved by the Clinical Research Ethics Committee of the Joint Chinese University of Hong Kong (CREC Ref. No: 2015.206). Briefly, infant faecal samples were obtained from four 9–15 months old infants (3–15 month is known as the microbiome developmental phase [25]) without any antibiotic or medicine intake at least three months prior to the collection. All the infants were delivered by natural birth and undertook solid food and breast milk simultaneously at the time of the experiments, giving possibly the highest microbial diversity (solid food only and breast-feeding only gave different diversity [25]). The faecal samples were collected in sample collection tubes diluted at 1:1 *v*/*v* with stabilizing buffer containing 30% glycerol (Thermo Scientific Pierce, Waltham, MA, USA, to maintain bacteria viability during cold storage) supplemented with 0.1% cysteine hydrochloride (a reducing agent to maintain anaerobicity). Faecal samples were stored in −20 °C during the delivery to the laboratory and were kept at −80 °C upon arrival. The viability of the faecal bacteria was checked by using total plate count (TPC) with reinforced clostridial broth (Oxoid Thermo, Waltham, MA, USA) with 2% agar (RCA) to ensure a sufficient number of viable bacteria at a dilution of 10^4^. Any faecal sample with a TPC of less than 300 Colony Forming Units (CFUs) at the dilution of 10^4^ was rejected. All faecal samples from the four infants were then pooled together with equal TPC CFU ratio to consolidate the generalizability of findings.

### 4.3. Depolymerization of Barley β-Glucans

Low viscosity barley β-1,3-1,4-glucans (BBGs) was obtained from Megazyme (product code: P-BGBL, >95% purity as stated). Acid hydrolysis was performed with different concentration of sulfuric acid (0.05, 0.10 and 0.20 M) for various time periods (10, 60, and 120 min) to obtain a range of BBG samples with different MWs. Briefly, an aqueous solution of BBG at a concentration of 10 mg/mL was prepared and maintained at 80 °C, followed by the addition of an appropriate amount of sulfuric acid (8M) to give a final concentration of 0.05, 0.1 and 0.2 M. The samples were stirred at 80 °C for the various time periods. The preparation scheme for BBG is shown in Table 1. A total of five BBG samples, including the native BBG and four depolymerized derivatives were obtained after the different acid hydrolysis conditions and they were denoted as BBG, BBG_0.05, BBG_0.1, BBG_0.2, and BBG_0.2(2). These sample solutions were neutralized with NaOH, dialyzed in Spectra/Por tubing with molecular weight cut off (MWCO) 3 kDa (Repligen, Waltham, MA, USA) for desalting in Milli-Q water for 24 h with three changes of water until the conductivity of the Milli-Q water reached single digit (Ciba-Corning Cond/TDS, Corning, NY, USA). All dialyzed BBG samples were then freeze-dried.

### 4.4. Chemical Composition and Linkage Analysis of BBGs

The total carbohydrate and total protein contents of the five BBG samples were determined by the phenol-sulfuric acid method and the bicinchoninic acid (BCA) protein assay (Thermo Scientific Pierce, Waltham, MA, USA), respectively by protocols described previously [26]. The monosaccharide composition of the native BBG and its depolymerized derivatives were determined by gas chromatographic analysis of the alditol acetate derivatives of the sugars formed by sequential acid hydrolysis, reduction and acetylation described previously [27]. The glycosidic linkages of the five BBG samples were determined by methylation analysis. In brief, partially methylated alditol acetate (PMAA) derivatives of the samples were prepared using CH_3_I and solid NaOH in dry DMSO described in a modified protocol reported previously. A gas chromatograph (6890N, Agilent Technologies, Santa Clara, CA, USA) coupled to a mass spectrometer (5973N, Agilent Technologies) (GC-MS) was used for analysis of both monosaccharides as their alditol acetate derivatives and linkage positions between sugar residues as their PMAA derivatives by protocols described previously [26].

### 4.5. Molecular Weight Distribution of BBGs

The MW distribution of the five BBG samples was determined using size exclusion chromatography (SEC) (Waters e2695 HPLC system) coupled with a refractive index (RI) detector (Waters 2414, Waters Inc., Milford, MA, USA) [28]. In brief, a size exclusion column TSK gel G5000 PW (30 cm × 7.5 mm i.d., Supelco, Bellefonte, PA, USA) was used to determine the MW profile. A range of dextran standards (5, 25, 80, 150, 410, 670 kDa) were used for calibrating the MW in the SEC analysis. The samples were dissolved in Milli-Q water to a concentration of 10 mg/mL and filtered through 0.22 mm nylon syringe filter before injection. Water was applied as mobile phase at a flow-rate of 0.7 mL/min at 30 °C.

### 4.6. Spectroscopic Methods of BBGs

The infrared spectra of the five BBG samples were recorded with a Fourier transform infrared spectrometer (FT-IR, Nicolet 670, Thermo Scientific, Waltham, MA, USA) in the range 4000–400/cm using the KBr-disk method [29]. One-dimensional proton NMR measurements of the samples were analyzed on a 700 MHz NMR spectrometer (Bruker Advanced III HD, Bruker, Billerica, MA, USA) at 60 °C using DMSO-d_6_ as the solvent [30].

### 4.7. In Vitro Deep Well-Plate Fermentation

Modified medium for colonic broth (mMCB) was used as the basal medium for the fermentation experiments as previously reported [29] and contained (per litre) 6.5 g of bacteriological peptone, 5.0 g of soy peptone, 2.5 g of tryptone, 3.0 g of yeast extract, 2.0 g of KCl, 0.2 g of NaHCO_3_, 4.5 g of NaCl, 0.5 g of MgSO_4_·7H_2_O, 0.45 g of CaCl_2_·2H_2_O, 0.2 g of MnSO_4_·H_2_O, 0.005 g of FeSO_4_·7H_2_O, 0.005 g of ZnSO_4_·7H_2_O, 0.4 g of cysteine–HCl, 0.005 g of hemin, 0.005 g of menadione, 0.5 mL of H_3_PO_4_, and 2 mL of Tween 80. No added carbon sources. All five BBG samples as well as glucose (as β-glucan monomer) were added as the sole carbon source at a concentration of 2% (*w*/*v*). Pooled infant faecal inoculum was mixed with mMCB having individual carbon sources at a volume ratio of 1:9 [31]. All fermentation mixtures (carbon sources in mMCB with infant faecal inoculum) were done in biological triplicates for all five BBGs and glucose monomer. Fermentation was conducted in deep well plates (Thermo Scientific) with each well composed of 2 mL of fermentation mixtures in an anaerobic jar containing AnaeroGen (Oxoid Thermo) and incubated at 37 °C for 40 h [31]. At the end of the fermentation period, aliquots of the fermentation samples (from the triplicates of each of the 6 carbon sources) were collected for microbial plating (100 µL), DNA extraction (200 µL), short chain fatty acid profiling (600 µL) and ammonia determination (100 µL).

### 4.8. Standard Bacterial Plate Count

Standard bacterial plate count was done using Reinforced Clostridia Broth (RCB) (Oxoid Thermo) plus 2% Bacteriological Agar (Oxoid Thermo), maintained under anaerobic conditions in duplicate using standard protocols of serial dilution with sterile PBS [31]. The growth of the total bacteria in the fermentation mixture after 40-h fermentation was expressed as CFUs after 48 h anaerobic incubation at 37 °C.

### 4.9. Short Chain Fatty Acids and Dissolved Ammonia Determination

SCFAs were extracted and analyzed using GC-FID with slight modifications [27]. In brief, 30 μL of methylvaleric acid (100 mg/mL) was added as internal standard and a hydrogen flow rate of 0.5 mL/min were used in the present method. A mixture of individual SCFA standards including acetic, propionic, butyric, valeric and caproic acid (SCFA standards kit, Alltech Inc., Nicolasville, KY, USA), and 4-methylvaleric acid (as internal standard) was prepared in 25% metaphosphoric acid at a final concentration of 10.0 mg/mL for identification and quantitation. The amount of the SCFAs after 24 h of fermentation was expressed in mM.

Ammonia was determined using Ammonia Rapid Assay (K-AMIAR, Megazyme). It is a highly specific assay involving the enzymatic conversion of 2-oxoglutarate, NADPH and dissolved ammonia into l-glutamic acid, NADP^+^ and water by glutamate dehydrogenase. Briefly according to manual, 100 µL fermentation samples were centrifuged at 14000 rpm (Spectrafuge 24D, Labnet, Woodbridge, NJ, USA) for 5 min to obtain supernatant, then 2 µL of samples, ammonia standard (0.04 mg/mL) and water as blank were added with 30 µL Buffer and 20 µL NADPH with 208 µL distilled water replaced by PBS pH 9.8 to maintain solubility of ammonia in reaction. Absorbance (A1) was taken at 340 nm with microplate reader (Molecular Devices SpectraMax Plus 384, San Jose, CA, USA). Then 2 µL glutamate dehydrogenase was added to each mixture and allowed to react for 5 min, followed by second absorbance (A2) at 340 nm. Calibration was also performed using a serial 2-fold dilution of ammonia standards. The final ammonia concentration (mg/mL) was calculated as: (Sample [A1 − A2]/Standard[A1 − A2]) × mg/mL Standard.

### 4.10. DNA Extraction, 16S Amplicon Sequencing and Bioinformatics

There was a total of 18 sequencing samples consisted of BBG, BBG_0.05, BBG_0.1, BBG_0.2, BBG_0.2(2), and glucose (each in biological triplicate). DNA extraction was conducted using EZNA Stool DNA Kits (Omega Bio-tek Inc., Norcross, GA, USA) in accordance with the manufacturer’s manual with some modifications [32]. These included an enzymatic pre-treatment step (mutanolysin and lysozyme) and a bead-beading step using a mix of 0.1 mm and 0.5 mm beads (Bioprep-24 Homogenizer & bead beater) to enhance DNA release from Gram positive bacteria. 16S amplicons were generated with Phusion polymerase (New England BioLabs, Ipswich, MA, USA) with barcoded forward primers targeting 16S V3 region [32] based on manufacturer’s protocols. After PCR, sharp DNA bands appeared at approximately 200 bp were cut and gel purified using QiaQuick Gel Extraction kit (Qiagen, Venlo, Netherlands) followed by AMPure treatment using the Agencourt AMPure XP system (Beckman Coulter, Brea, CA, USA). The quantity and quality of DNA after AMPure treatment were accessed using a Qubit Fluorometer (Thermo Scientific, Waltham, MA, USA) and Bio-Analyzer (Agilent, Santa Clara, CA, USA). Sequencing library was prepared by pooling the 18 samples in equal molar ratio. Then, emulsion PCR and enrichment were done using the Ion PGM Template OT2 400 Kit in Ion OneTouch 2 System (Thermo Scientific, Waltham, MA, USA). The DNA sequencing was done using the Ion PGM Sequencing 400 Kit on Ion torrent PGM using Chip 318v2 in the Core Facilities of the Chinese University of Hong Kong. After sequencing, the individual sequence reads were filtered by the PGM software to remove low quality and polyclonal sequences. Sequences matching the PGM 3′ adaptor were also automatically trimmed. All PGM quality-approved, trimmed and filtered data were exported as .sff files. Bioinformatics analysis was done using Qiime-v1.9.1 pipeline [33]. Greengenes-v13.8 was used as reference for bacterial 16S sequence database [34]. Sequences were trimmed for primers and barcodes. The cleaned sequences were then clustered at 97% similarity as Operational Taxonomic Unites (OTUs) followed by deletion of chimeras and singleton reads. α and β diversity were then analyzed. KEGG prediction was carried out using PICRUSt-v1.0.0 [14] with NSTI score output included and statistically analyzed using STAMP-v2.1.3 [35]. Bacterial biomarkers unique to groups were identified using LEfSe [36]. Significant microbial taxa were also identified using LEfSe. High level microbial phenotypes that could not predicted by PICRUSt were analyzed using BugBase and default BugBase traits were predicted [37]. All computational analysis was carried out on a local Dell Workstation with Quad Core Intel^®^ Xeon^®^ Processor and 32G RAM.

### 4.11. Integration and Statistical Analysis

Integration analysis was done by fitting the trends of different parameters (DNA amount/terminal glucose/SCFAs/NH3/microbial taxa) investigated in this study against increase in MW modelled with regression equation and coefficient of determination provided. Unless otherwise specified, R software-v3.5.2 [38] was used for statistical calculation and independent pairwise student’s t-test or ANOVA with Tukey HSD post-hoc adjustment (CI = 0.95) was used. Statistical test parameters for bioinformatics tools include: STAMP (Statistical test: ANOVA with Tukey-Kramer post-hoc test CI = 0.95, multiple test correction: Benjamini-Hochberg FDR); LEfSe (alpha = 0.05 for factorial Kruskal-Wallis test; alpha = 0.05 for pairwise Wilcoxon test; threshold = 2.0 for logarithmic LDA score) and BugBase (threshold values were automatically defined with highest variance across all sample, output to be <0.03, ANOVA with Tukey HSD post-hoc adjustment (CI = 0.95) was used).

## 5. Conclusions

Our present results have demonstrated a partial acid hydrolysis under controlled conditions seemed to be an effective means to depolymerize BBG into derivatives with lower MW. It is a versatile method suitable for screening and purification after hydrolysis. We have also demonstrated that the platform used for infant faecal sample collection and storage as well as using the deep well plate to serving as multiple miniature bioreactors, is applicable for in vitro fermentation. We have also coined the SCFAs/NH_3_ ratio as a new indicator of eubiosis/dysbiosis in terms of saccharolytic and proteolytic fermentation. Correlation analysis had shown that most of the fermentation parameters investigated showed a parabolic function instead of a linear function with the BBG samples having different MW. Microbial community investigation is challenging as there is a myriad of interactions among all the microbes. We have exemplified this idea with the monotonous pattern, U-shape pattern, inverted-U shape pattern, wave-like pattern of some selected taxa. Among the five BBGs, we concluded that the BBG_0.1 with intermediate MW of 28 kDa showed the most promising lead to be developed as a potential novel prebiotic. The data of this project has been submitted to NCBI SRA and available under the BioProject ID: PRJNA487303, SRA accession: SRP158602.

## Figures and Tables

**Table 1:** Preparation and structural characteristics of barley β-glucans (BBG) and its acid hydrolyzed derivatives.

Samples *	Carbohydrate (% by wt)	Protein (% by wt)	Molecular Weight (kDa)
BBG	98.0 ± 0.7	0.2 ± 0.5	319
BBG_0.05	98.2 ± 0.3	0.2 ± 0.7	104
BBG_0.1	97.6 ± 0.3	0.2 ± 0.1	28
BBG_0.2	98.1 ± 0.5	0.2 ± 0.3	11
BBG_0.2(2)	97.9 ± 0.4	0.2 ± 0.1	6

* The preparation conditions were: BBG: original, no hydrolysis; BBG_0.05: 0.05M H_2_SO_4_, 10 min at 80 °C; BBG_0.1: 0.10M H_2_SO_4_, 60 min at 80 °C; BBG_0.2: 0.20M H_2_SO_4_, 60 min at 80 °C; BBG_0.2(2): 0.20M H_2_SO_4_, 120 min at 80 °C.

**Table 2:** GC-MS linkage analysis of barley β-glucan (BBG) and its acid hydrolyzed derivatives.

Samples	Peak Area (%) ^a^			Relative Molar Ratio ^c^ T-Glc*p*:1,3-Glc*p*:1,4-Glc*p*	Relative Molar Ratio ^c^ T-Glc*p*:1,3-Glc*p*:1,4-Glc*p*
	T-Glc*p* ^b^	1,3-Glc*p*	1,4-Glc*p*		
BBG	3.99 ± 1.00	14.25 ± 0.15	81.76 ± 0.85	**1**:3.57:20.49	0.28:**1**:5.74
BBG_0.05	3.76 ± 0.94	14.15 ± 0.14	82.09 ± 0.80	**1**:3.77:21.84	0.27:**1**:5.80
BBG_0.1	13.22 ± 2.99	13.11 ± 0.45	73.68 ± 2.54	**1**:0.99:5.58	1.01:**1**:5.62
BBG_0.2	22.46 ± 4.55	9.94 ± 0.58	67.60 ± 3.97	**1**:0.44:3.01	2.26:**1**:6.80
BBG_0.2(2)	32.28 ± 5.73	9.24 ± 0.78	58.48 ± 4.94	**1**:0.29:1.81	3.49:**1**:6.33

^a^: calculated from the peak area in the GC-MS total ion chromatograms. ^b^: Glc*p*, glucopyranose. ^c^: molar ratio was calculated from the ratio of the (peak area)/(MW) of individual PMAAs.

**Table 3:** Bacterial total plate count (CFUs) and concentration of total DNA extracted from culture broth of the infant faecal fermentation of five BBG samples, glucose monomer and time-0.

Samples	Total Plate Count (CFU)	Ratio of CFUS (Relative to Glucose)	[DNA] (µg/mL) *	Ratio of [DNA] (Relative to Glucose)
BBG	8.7 × 104	3.7	67.3 ± 7.4 ^a,b^	3.4
BBG_0.05	2.3 × 104	1.0	76.4 ± 4.0 ^c,d^	3.9
BBG_0.1	5.4 × 104	2.3	83.5 ± 9.1 ^a,e,f^	4.2
BBG_0.2	6.8 × 104	2.9	71.9 ± 4.1 ^g,h^	3.6
BBG_0.2(2)	5.2 × 104	2.2	54.8 ± 0.6 ^c,g,i^	2.8
GLC	2.4 × 104	1	19.7 ± 2.4 ^b,d,e,f,h,i^	1
T0	6.4 × 103	0.3	4.3 ± 0.2 ^@^	0.2

* Mean values with different superscripts (^a–i^) represent significant difference by one-way ANOVA (Tukey HSD post-hoc test), *p* < 0.05. ^@^: Time-0, T0 group is statistically significant different compared to all other groups.

**Table 4:** Rarefied α-diversity indices of the infant faecal fermentation of 5 BBG samples and glucose monomer.

Samples	Phylogeny-Based	Non-Phylogeny-Based				
	PD Whole Tree	Fisher Alpha	Berger Parker D	Shannon	Simpson_E	Simpson Reciprocal
**BBG**	17.68 ± 0.77 ^a,b^	90.81 ± 4.66 ^a,b^c	0.18 ± 0.01 ^a,b,c,d,e^	4.87 ± 0.08 ^a,b,c,d^	0.026 ± 0.001 ^a^	13.17 ± 0.84 ^a,b,c,d^
**BBG_0.05**	18.69 ± 0.50	100.40 ± 1.89	0.14 ± 0.01 ^a,f^	5.22 ± 0.07 ^a,e^	0.031 ± 0.002 ^b^	16.96 ± 0.92 ^a,e^
**BBG_0.1**	19.95 ± 0.59 ^a^	103.76 ± 5.26 ^a^	0.15 ± 0.01 ^b,g^	5.31 ± 0.11 ^b,f^	0.033 ± 0.001 ^a,c^	18.27 ± 1.15 ^b,f^
**BBG_0.2**	19.41 ± 0.95	105.66 ± 2.98 ^b^	0.13 ± 0.01 ^c,h^	5.21 ± 0.04 ^c,g^	0.031 ± 0.000 ^d^	17.69 ± 0.51 ^c,g^
**BBG_0.2(2)**	19.85 ± 0.53 ^b^	107.50 ± 5.94 ^c^	0.15 ± 0.01 ^d,i^	5.12 ± 0.08 ^d^	0.028 ± 0.004	16.14 ± 1.50 ^d,h^
**GLC**	19.34 ± 0.81	101.11 ± 5.89	0.22 ± 0.01 ^e,f,g,h,i^	4.93 ± 0.02 ^e,f,g^	0.023 ± 0.002 ^b,c,d^	12.63 ± 0.25 ^e,f,g,h^

^a–i^ Different superscripts represent significant difference by one-way ANOVA (Tukey HSD post-hoc test), *p* < 0.05.

## Supplementary Materials

The following are available online, Figure S1, (**A**) GC monosaccharide profile; (**B**) FTIR spectrum; and (**C**) NMR 1H spectrum of BBG and BBG_0.2(2). Figure S2, Representative bacterial total plate count of the 5 BBG samples and glucose monomer. Figure S3, GC-FID chromatograms (**A**) Short chain fatty acid external standards, C2, C3, C4, C5, C6 and internal standard, methyl-valeric acid; (**B**) short chain fatty acid profiles of the BBG after 40 h of fermentation. **Figure S4**, Overall distribution of metagenome KEGG prediction output of the infant faecal fermentation of 5 BBG samples, glucose monomer and T0 group using PICRUSt. (**A**) KEGG level 1 class distribution; (**B**) expanded KEGG level 2 class distribution from level 1 “Metabolism” group. Figure S5, Selected metagenome KEGG prediction output of the infant faecal fermentation of 5 BBG samples and glucose monomer using PICRUSt and statistically analysis using STAMP. (**A**) Carbohydrate metabolism; (**B**) ABC transporters; (**C**) Bacterial secretion system; and (**D**) Glycan biosynthesis and metabolism. Figure S6, Selected metagenome KEGG prediction output of the infant faecal fermentation of 5 BBG samples and glucose monomer using PICRUSt and statistically analysis using STAMP. (**A**) Lipid metabolism; (**B**) Protein digestion and absorption; (**C**) Amino acid metabolism; and (**D**) Nitrogen metabolism. Table S1, All the identified microbial taxa of the 5 BBGs (XLSX format). Table S2, The output of PICRUSt metagenome prediction of infant faecal fermentation of 5 BBG samples and glucose (XLSX format). Table S3, The output of LEfSe of infant faecal fermentation of 5 BBGs and glucose (XLSX format).
