# Supplementary material for: In Vitro Infant Faecal Fermentation of Low Viscosity Barley β-Glucan and Its Acid Hydrolyzed Derivatives: Evaluation of Their Potential as Novel Prebiotics
Source: Molecules. 2019 Feb 26;24(5):828. doi: 10.3390/molecules24050828 (PMC6429510; doi:10.3390/molecules24050828)
Supplement: Supplementary file 1 [file molecules-24-00828-s001.zip › Final SM/Final SM.pdf]

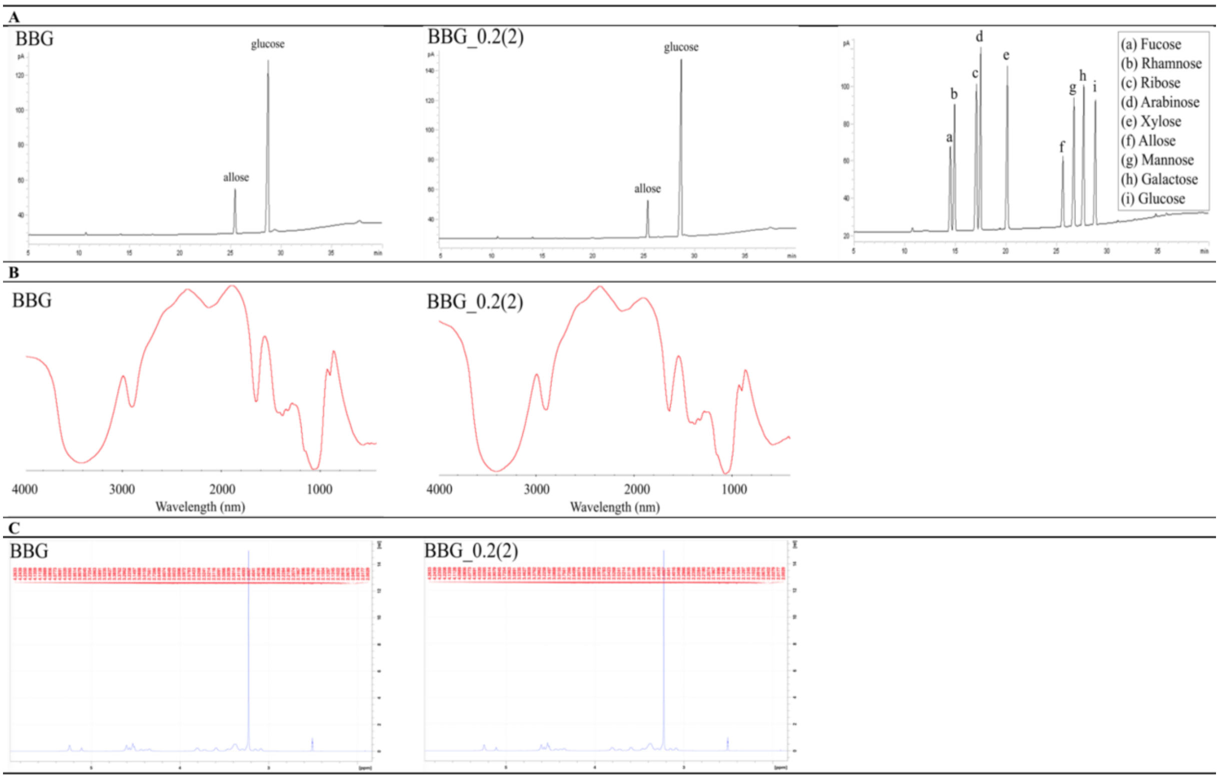

Figure 1. (A) GC monosaccharide profile; (B) FTIR spectrum; and (C) NMR 1H spectrum of BBG and BBG_0.2(2).

Samples	Representative plate of fermentation mixture after 40 h	Samples	Representative plate of fermentation mixture after 40 h
BBG	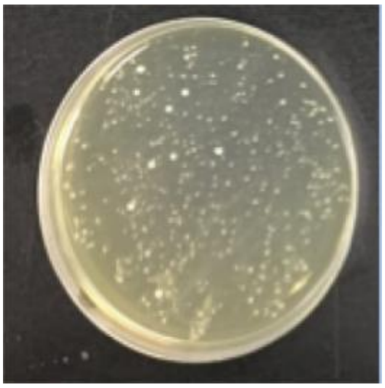	BBG_0.2	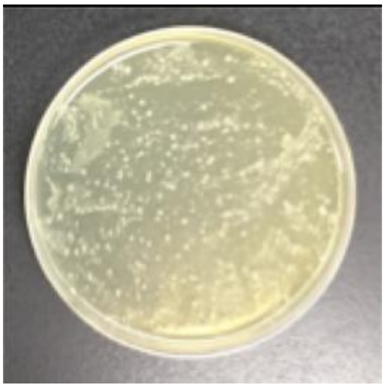
BBG_0.05	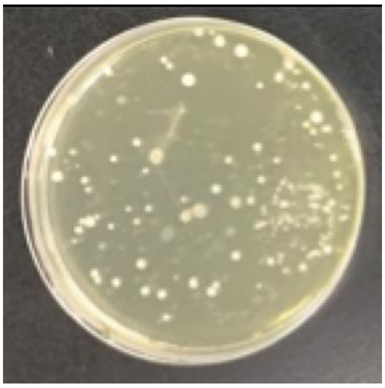	BBG_0.2(2)	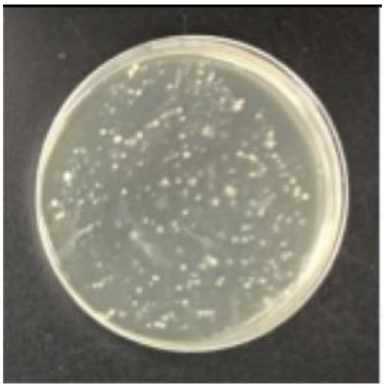
BBG_0.1	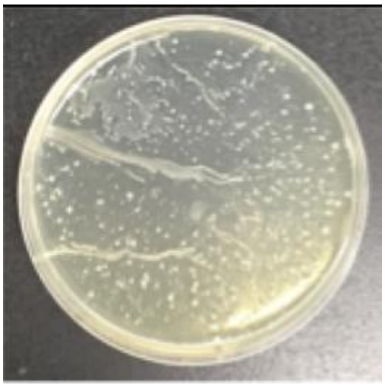	Glucose	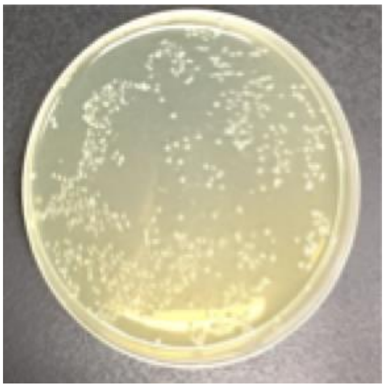

Figure 2. Representative bacterial total plate count of the 5 BBG samples and glucose monomer.

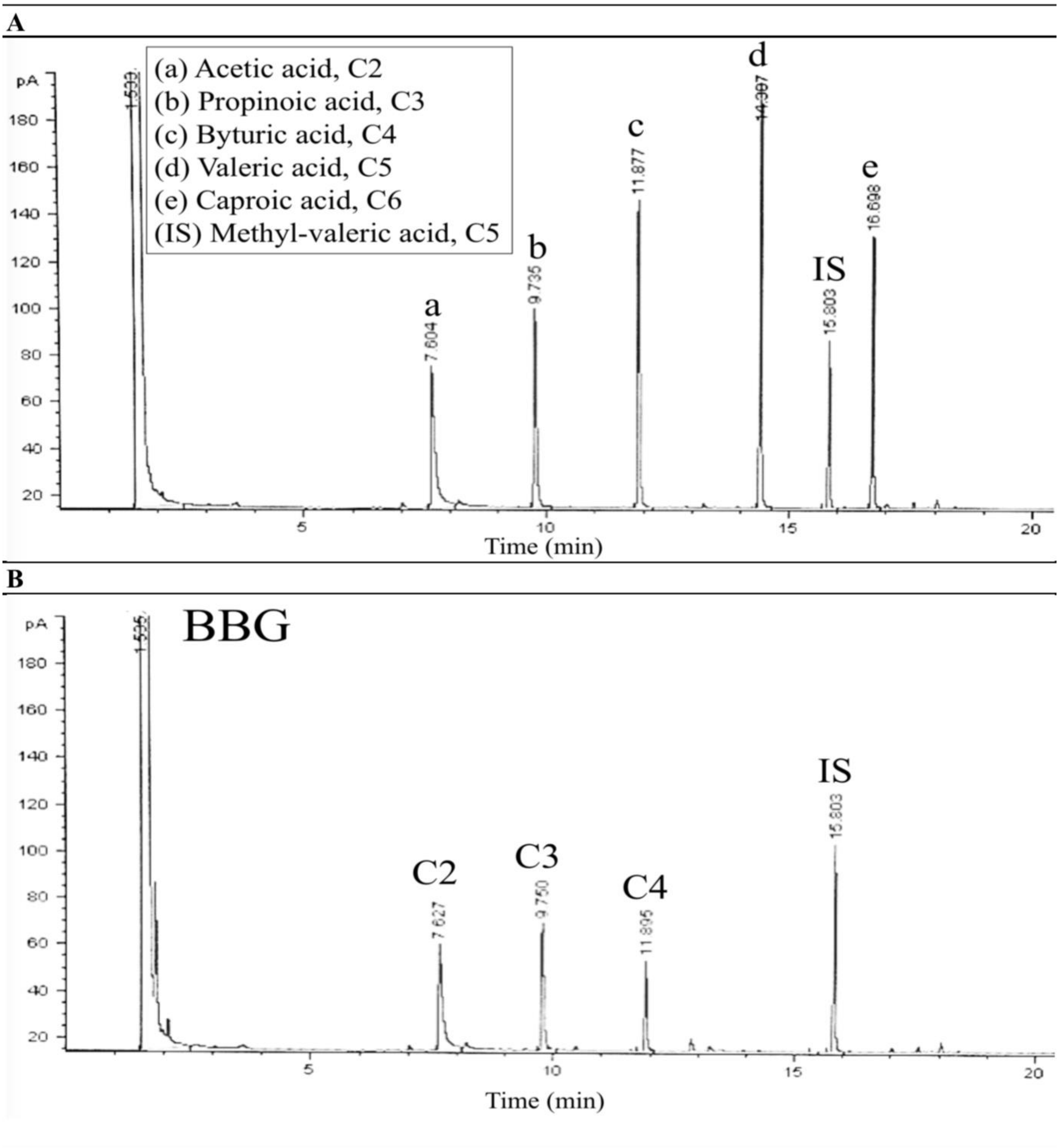

Figure 3. GC-FID chromatograms (A) Short chain fatty acid external standards, C2, C3, C4, C5, C6 and internal standard, methyl-valeric acid; (B) short chain fatty acid profiles of the BBG after 40 h of fermentation.

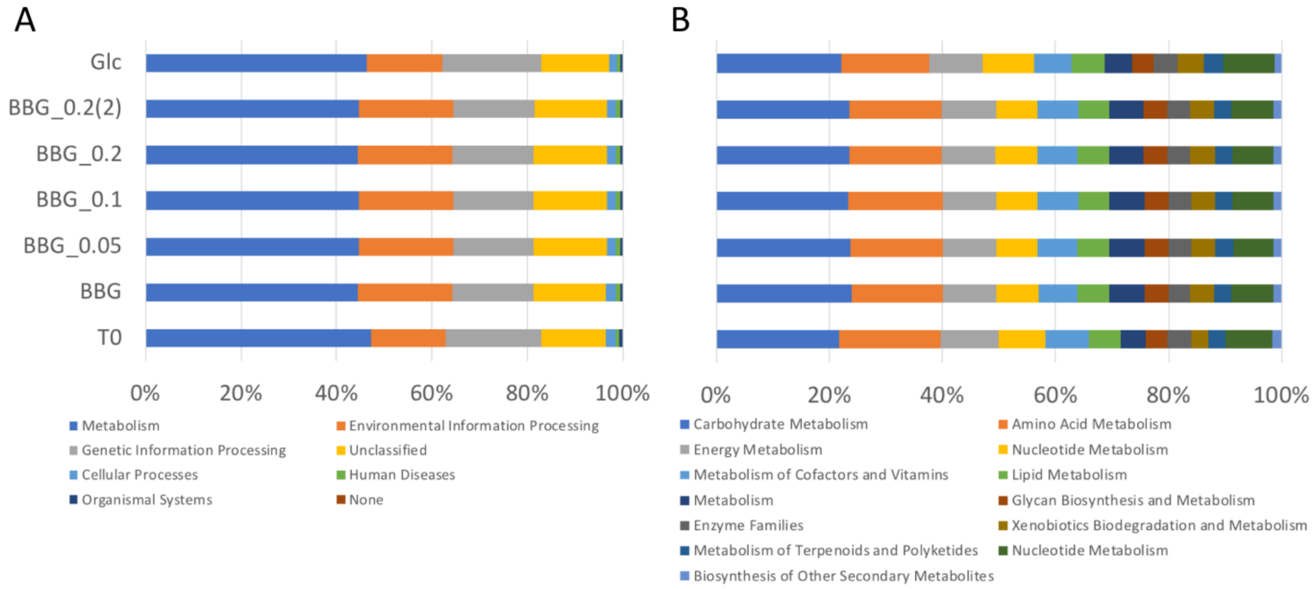

Figure 4. Overall distribution of metagenome KEGG prediction output of the infant fecal fermentation of 5 BBG samples, glucose monomer and T0 group using PICRUSt. (A) KEGG level 1 class distribution; (B) expanded KEGG level 2 class distribution from level 1 "Metabolism" group.

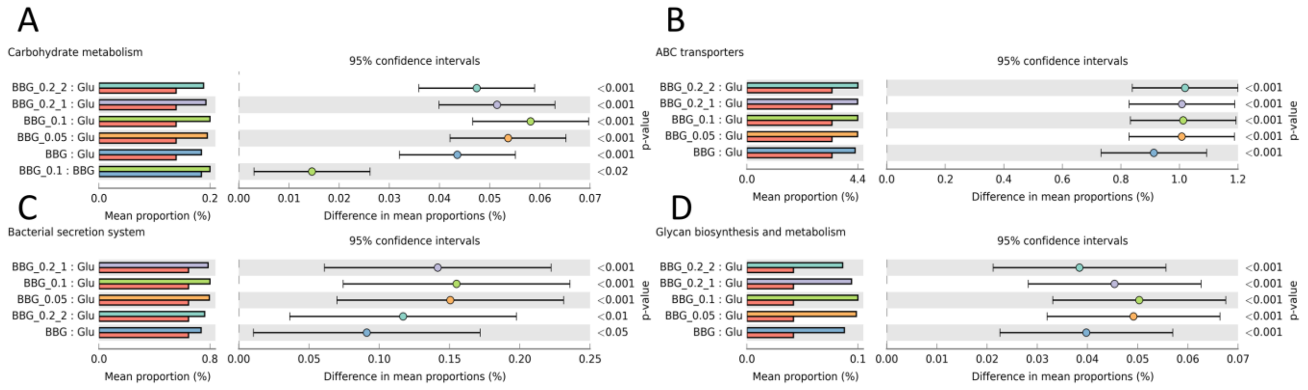

Figure 5. Selected metagenome KEGG prediction output of the infant fecal fermentation of 5 BBG samples and glucose monomer using PICRUSt and statistically analysis using STAMP. (A) Carbohydrate metabolism; (B) ABC transporters; (C) Bacterial secretion system; and (D) Glycan biosynthesis and metabolism.

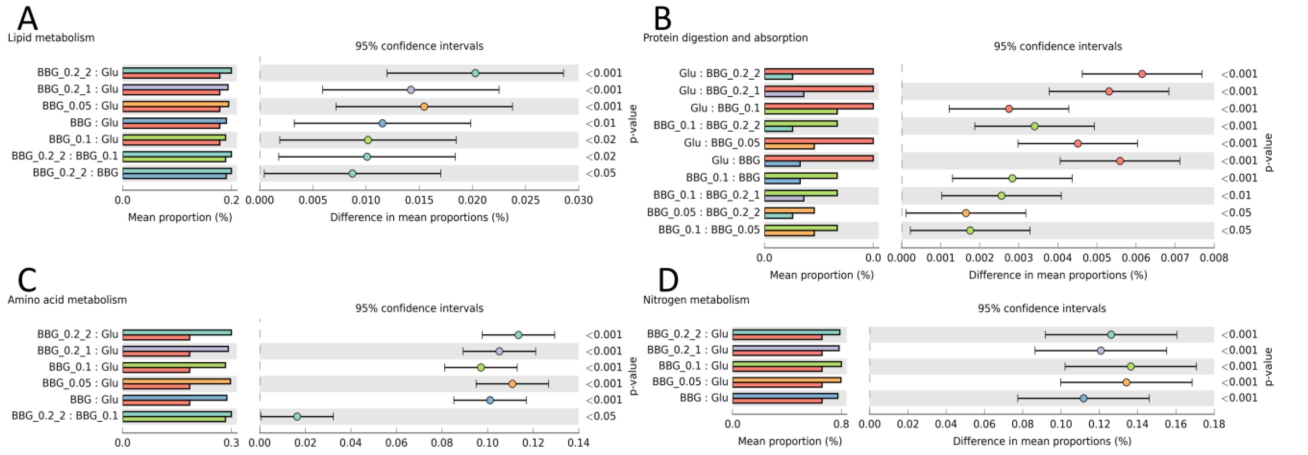

Figure 6. Selected metagenome KEGG prediction output of the infant fecal fermentation of 5 BBG samples and glucose monomer using PICRUSt and statistically analysis using STAMP. (A) Lipid

metabolism; (B) Protein digestion and absorption; (C) Amino acid metabolism; and (D) Nitrogen metabolism.
